# A Study on Improving the Mechanical Performance of Carbon-Fiber-Reinforced Cement

**DOI:** 10.3390/ma12172715

**Published:** 2019-08-24

**Authors:** Yeou-Fong Li, Tzu-Hsien Yang, Chang-Yu Kuo, Ying-Kuan Tsai

**Affiliations:** 1 Department of Civil Engineering, National Taipei University of Technology, 1, Sec. 3, Chung-Hsiao E. Rd., Taipei 10608, Taiwan; 2 Department of Materials and Mineral Resources Engineering, National Taipei University of Technology, 1, Sec. 3, Chung-Hsiao E. Rd., Taipei 10608, Taiwan; 3 Department of Environmental Information and Engineering, Chung Cheng Institute of Technology, National Defense University, PO Box 90047-82, Dasi, Taoyuan, Taiwan

**Keywords:** short carbon fiber, silane, early-strength cement, pneumatic dispersion, high temperature

## Abstract

This study investigated several approaches for silane-removal from the surface of short carbon fiber bundles, and short carbon fibers uniformly dispersed in cement to produce a novel compound of carbon-fiber-reinforced cement. In order to facilitate the uniform distribution of short carbon fibers in the carbon-fiber-reinforced cement, it is necessary to remove the silane from the carbon fiber’s surface. Short carbon fiber bundles were submerged into a pure water, sodium hydroxide solution, and acetic acid solution, and placed in high-temperature furnace used to remove silane from the carbon fiber surface. The results were observed under a scanning electron microscope to determine the level of silane removal from the surface, and an effective method for removing the silane was developed from among the several approaches. This method employed a pneumatic dispersion device to disperse carbon fibers then mixed in a high-early-strength cement which led to an excellent compressive and impact-resistance performance of carbon-fiber-reinforced cement. Final testing showed that the compressive strength and impact energy increased by 14.1% and 145%, respectively.

## 1. Introduction

Civil and military reinforced concrete infrastructures often suffer from impacts and loading of high instantaneous energy that could cause infrastructures damage. Fiber reinforced cement, mortar, and concrete are used to repair or rebuild damaged reinforced concrete infrastructure. However, the fibers in the cement, mortar, or concrete sometime cannot be uniformly distributed. Recently, the price of the carbon fiber is much less than that in last two decades; therefore, the carbon fiber was widely used in sporting goods, automotive, aerospace, and civil engineering. This paper aims to improve the mechanical properties of the carbon-fiber-reinforced high-early-strength cement (carbon-fiber-reinforced cement) by using several approaches for silane-removal from the surface of short carbon fiber bundle and using pneumatic disperser to disperse the short carbon fibers.

The spinning process of commercial carbon fibers involves adding an appropriate amount of silane to produce yarn bundles for weaving. Silanes are widely used in industry as a coupling agent, which possesses differing functional groups at both molecular ends. A coupling agent, with one of its ends bonding with inorganic substances and the other end bonding with organic polymers, bonds tightly between organic and inorganic interfaces and improves the compatibility and adhesion between two materials. In the production of fiber-reinforced concrete, when unprocessed short carbon fiber bundles are mixed into the cement, non-uniform distribution of short carbon fiber strands can occur in the cement. To facilitate uniform distribution of collaborated short carbon fibers in the cement, the silane must be removed from the carbon fiber surfaces to strengthen the bonding between the carbon fibers and the cement. The silanes can be successfully removed from the surfaces of the carbon fiber by performing hydrolysis, which involves diluting and dissolving the silanes in isopropanol alcohol or water, immersing the silanes in acid or alkali solutions, and heating the carbon fibers higher than the boiling point of silane using a high-temperature furnace. In particular, the pH level for the hydrolysis (activation) reaction of the silanes is 2–6. Factors influencing the hydrolysis are silane concentration, pH level, temperature, humidity, and solvents, and a larger size of alkoxy groups indicates lower hydrolysis rates of the silanes [1,2]. Furthermore, H^+^ and OH^−^ ions in solutions can be used as catalysts to accelerate the hydrolysis of the silanes.

Generally, the carbon fibers are added into Portland cement to increase the bending resistance and toughness of the cement; however, because the properties are related to the distribution, length, and additional amount of fiber, determining the appropriate amounts and lengths of fibers according to various construction characteristics can improve the performance of fiber-reinforced cementitious materials [3,4,5,6,7]. More uniform distribution of carbon fibers in cementitious materials yields higher effectiveness of the aforementioned properties. The distribution in the cement can be evaluated using statistical approaches and electron microscopy. Furthermore, several research projects defined the dispersion coefficient of carbon fiber to evaluate the dispersion of fibers in the cement composites [8,9,10]. The mechanical characteristics of the cement composites depend on not only the tensile strength of the fiber, but also the load transmittance between the fiber and the cement matrix. The excellent adhesion and dispersion of carbon fiber in the cement paste enhanced the flexural strength and improved the transfer of stress through the interface between the cement and carbon fiber [9,10,11,12,13,14,15,16]. The mixing approaches of fibers into cementitious composites are another factor affecting the dispersion. Most studies revealed that a premixing approach created a more uniform distribution of fibers in cementitious materials than a post-mixing approach [17,18,19,20,21]. Additionally, adopting the physical process like ultrasonic waves or vibration can improve the dispersion of fiber in concrete. In the most related studies, the effect of the microstructure of carbon fiber reinforced concrete (CFRC) on their macrostructure and mechanical properties of CFRC were investigated [22,23,24,25].

However, structures often suffer from impacts and loading of high instantaneous energy affecting the structural and concrete elements and members. This energy is transferred to the structures through different dynamic loads in an extremely short period of time or transmitted through high projectile mass with low impact velocity, both of which could cause significant structural damage and even failure. Combining fiber and concrete can effectively enhance the ability to absorb dynamic energy of impact and promote mechanical performance. In contrast with other types of fiber, carbon fiber features higher strength, high-temperature resistance, corrosion resistance, better electrical conductivity, and lower thermal expansion coefficient. Thus, CFRC are commonly applied in civil engineering.

The present study utilized the surface treatment of short carbon fibers using various approaches and observed the scanning electron microscope (SEM) images to examine the effectiveness of silane removal in each approach. Finally, the carbon-fiber-reinforced cement specimens were added short carbon fibers with and without residual silane, then compared the differences between the presence and absence of residual silane regarding their influences on the compressive and impact strengths of carbon-fiber-reinforced cement.

## 2. Materials 

In carbon-fiber-reinforced cement, the cement provides the overall strength and carbon fibers provide the toughness of the cement. Carbon fibers exhibit high tensile strength, and the bonding strength between carbon fibers and cement increases the toughness of carbon-fiber-reinforced cement. This study produced carbon-fiber-reinforced cement using high-performance of polyacrylonitrile (PAN)-based carbon fibers additive added to mix with the high-early-strength cement. 

### 2.1. Carbon Fiber

**Figure 1:**
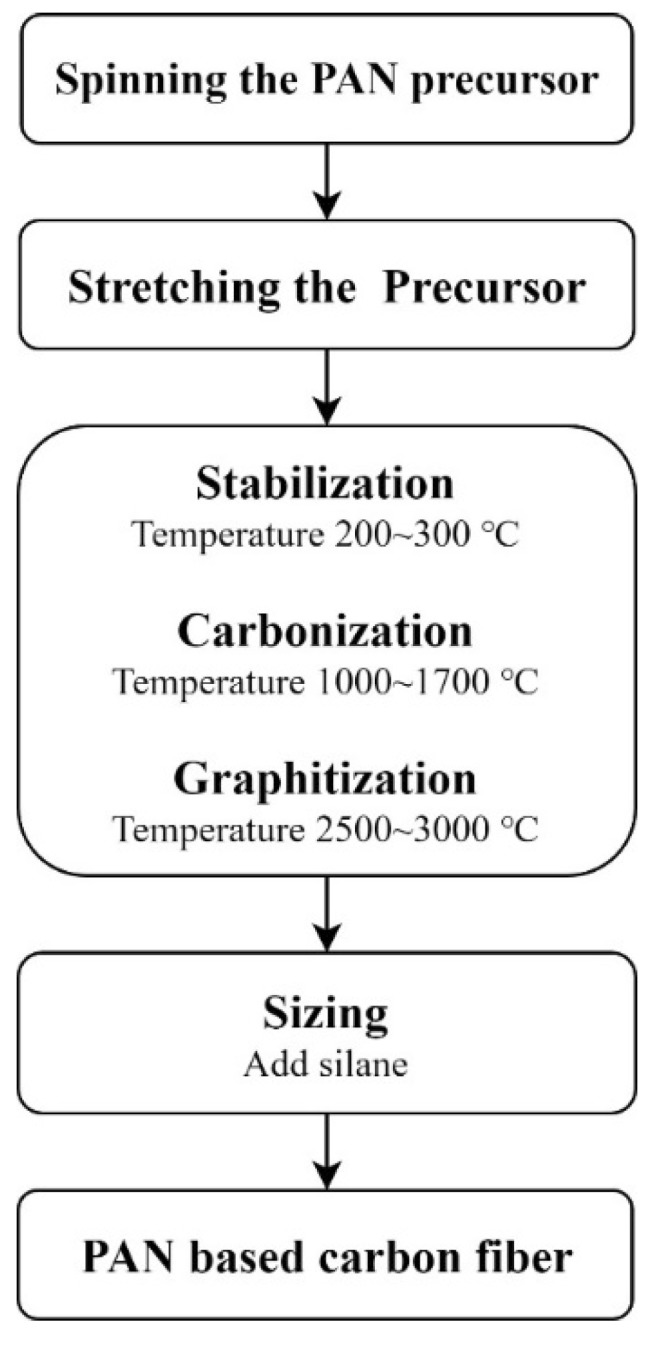
High-performance, polyacrylonitrile (PAN)-based carbon fiber production process [26].

The short carbon fibers used in this study were high-performance, PAN-based carbon fibers, and their production process is as follows: crude oil was refined and cracked into propylene (PP) and the refined oil was reacted with NH_3_ to produce acrylonitrile by spinning the PAN precursor followed by stabilization, carbonization, and graphitization under high temperatures. The production process is shown in Figure 1 [26].

This study used short carbon fibers manufactured by Tairylan Division, Formosa Plastics Group, to produce the carbon-fiber-reinforced cement. Each bundle of the short carbon fibers comprises approximately 12,000 carbon fiber strands, and each strand has a length of 24 mm, density of 1.81 g/cm^3^, diameter of 7.0 μm, elongation rate of 2.0%, tensile strength of 4.9 GPa, and elastic modulus of 250 GPa.

### 2.2. High-Early-Strength cement

The carbon-fiber-reinforced cement had high-early-strength manufactured by Denka Company. Finer cement exhibits a higher shrinkage and a greater proneness to cracking. Portland cement hardening and strength much depend on the composition of the processed clinker. Strength properties are known to much depend on the tricalcium silicate (C_3_S) content. The precondition of high early strength is the presence of the mineral component C_3_S in proper quantity. High-early-strength cement is nothing but still finer cement with high C_3_S and low gypsum content. As increasing the content of C_3_S in the cement and the fineness of the cement, its hydration speeds up in early stages, and its early strength increases. With an appropriate water-cement ratio, the cement could achieve a strength of 28 MPa and a slump of 4~10 cm at an age of 1 day. By performing X-ray fluorescence analysis, the major composition consisted of oxides. All components in the high-early-strength cement are analyzed in Table 1. 

## 3. Methods

The present study utilized the surface treatment of short carbon fibers using various approaches and observed the SEM images to examine the effectiveness of silane removal in each approach. Finally, short carbon fibers, with and without residual silane enclosed, then compared the differences between the presence and absence of residual silane regarding their influences on the compressive and impact strengths of carbon-fiber-reinforced cement.

### 3.1. GC-MS Testing

Gas Chromatography and Mass Spectrometry (GC-MS) are techniques for the analysis and quantification of organic volatile and semi-volatile compounds. The GC-MS testing involved an initial temperature of 40 °C, which was increased temperature at a rate of 5 °C/min and an equilibrium time of 0.5 min, the final temperature set to 550 °C. The carbon fibers were immersed in pure water for 1 day before removing them and performing a GC-MS testing on the immersion solution to identify the type and boiling range of silane and other substances.

### 3.2. SEM Analysis

To evaluate the effectiveness of the silane removal from the carbon fibers, the experiment conducted an SEM observation on the surfaces of the short carbon fibers treated with various approaches. A high-resolution thermal-field-emission scanning electron microscope (model: JSM-7610F, JEOL, Tokyo, Japan) was used to perform the testing. The device had an accelerating voltage of 0.1–30 kV, an electronic light source in the form of an in-lens Schottky field emission electron gun, and secondary electron-imaging resolutions of 1.0 nm at 15 kV and 1.3 nm at 1 kV. Different approaches for removing the silane from the short carbon fiber surfaces yielded different effectiveness, which was evaluated using SEM observation to find if the residual silane was still remaining.

### 3.3. Compressive Testing

This study conducted compressive testing in accordance with the ASTM C 109/C 109M-02 standards, which entailed placing specimens in a universal testing machine and achieving a uniform contact between the machine and each specimen at a loading speed of 900–1800 N/s to obtain the maximum compressive strength of each specimen [27]. The specimen production involved removing the silane using the various methods, separating the carbon fibers using pneumatic dispersion, and mixing the spread short carbon fibers with cement evenly in a mixer. The mixing process comprised dry mixing for 120 s followed by wet mixing for 120 s with water added. The produced carbon-fiber-reinforced cement specimens were 5 × 5 × 5 cm^3^ cubes with carbon fibers that were 24 mm in length, with a weight percentage of added short carbon fibers of 1.0 % (0.8% in volume percentage), and a water–cement ratio of 0.31.

### 3.4. Impact Testing

**Figure 2:**
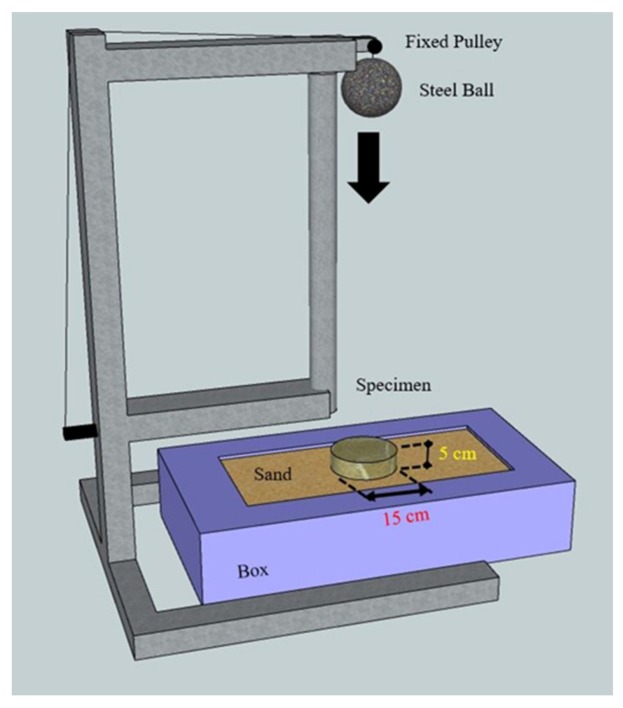
The illustration configuration of the impact test instruments.

In accordance with the ASTM D5628 standards, an impact testing was conducted [28]. This testing was aimed at examining the impact energy of load on flat cylindrical specimens with a diameter of 15 cm and a thickness of 5 cm. A steel ball was suspended using a string to a designated height before the string was released, causing the steel ball to free-fall onto the specimens. Subsequently, we observed the impact damage on the specimens by the steel ball; the settings for the impact testing are depicted in Figure 2. The experimental parameters comprised the weights of different steel balls and different falling heights for impact testing.

The impact testing included single and repeated impact testing. The single impact testing involved damaging specimens through a single free-fall drop and comparing their impact resistance according to the maximum impact energy after sustaining impact testing. By contrast, the repeated impact testing adopted a falling ball with a fixed weight and entailed continuously impacting the surface of specimens repeatedly from a certain height, and to record impact numbers of contacting of the steel ball and cement block. The impact test evolution was recorded by a high speed camera.

In the impact testing, the impact velocity, rebound velocity, and duration of contact time for collision between falling ball and specimen is captured and calculated through the software used in high-speed camera. The interval between the moment when a free-fall ball touched and left the surface of specimen is called contact duration (∆*t*). Then, according to the impulse momentum principle, we could calculate the applied force. The impulse-momentum formula is expressed as follows:

(1) m×Δv=F×Δt

where

*m*: mass 

∆*v*: variation from velocity of steel ball during contact duration 

*F*: force applied on specimen 

∆*t*: duration for forced applied on specimen 

The impact testing was conducted through high-speed camera frames, which could calculate the contact time and forced applied on the specimens by Equation (1). A high-speed camera (model: FASTCAM Mini UX100 type 200K-C-16G, IN, USA) was used in the impact test and the software adopted 10,000 fps and 1280 × 480 resolution. 

In the impact testing, the potential energy can be calculated by Equation (2):

(2) E=m×g×h

where

*E*: gravitational potential energy (J)

*m*: mass (kg)

*g*: gravitational acceleration (m/s^2^)

*h*: height (m)

## 4. Results and Discussion

**Figure 3:**
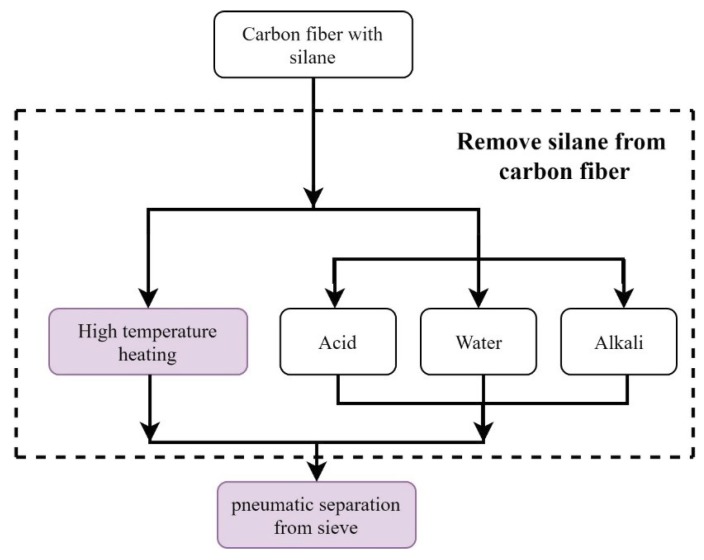
The flowchart of silane removal from short carbon fiber surfaces using different approaches.

**Figure 4:**
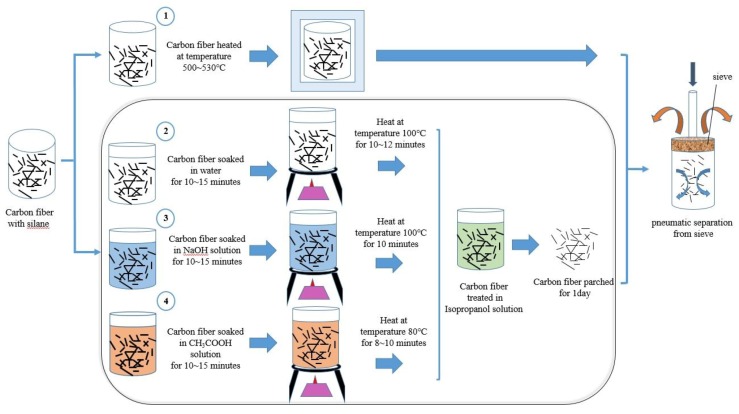
Illustration of silane removal from short carbon fiber surfaces using different approaches.

First, this study tested the type and boiling point of the silane on the carbon fiber surfaces and identified other substances contained in the carbon fibers. Subsequently, the silane on the surface of the carbon fibers was removed using chemical and physical vaporization approaches. The chemical solution approaches included immersing the carbon fibers in pure water, sodium hydroxide solutions, and acetic acid solutions; the physical approaches included heating the carbon fibers using a high-temperature furnace. Figure 3 depicts the flow chart of the silane removal process, and Figure 4 demonstrates each silane removal approach. In the distribution testing described in the following subsection, short carbon fibers weighing 10 g and with a length of 24 mm were used.

### 4.1. GC-MS testing

**Figure 5:**
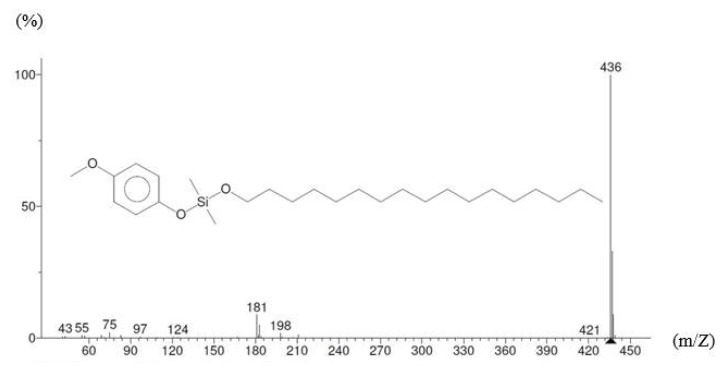
Silane and its molecular mass detected using a Gas Chromatography and Mass Spectrometry (GC-MS) test.

**Figure 6:**
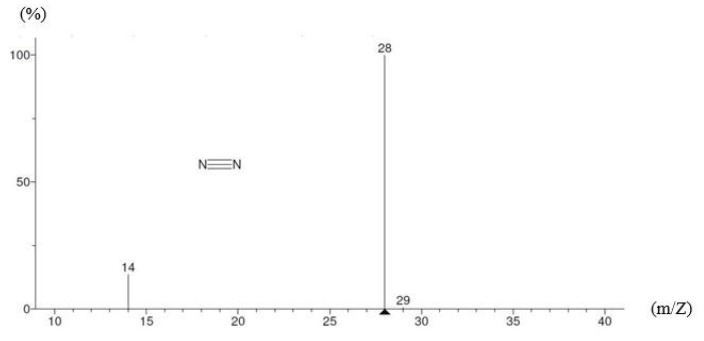
Substances and their molecular masses using a GC-MS test.

The carbon fibers immersed in pure water for 1 day before removing them and performing a GC-MS testing on the immersion solution to identify the type and boiling range of silane and other substances. The GC-MS testing involved an initial temperature of 40 °C, which was increased to 280 °C at a rate of 5 °C/min and a temperature equilibrium time of 0.5 min. At 47 min 20 s of heating, the study observed the precipitation of the silane and conducted mass spectrometry to determine its molecular structure (Figure 5); it was found to have a chemical formula of C_26_H_48_O_3_Si, molecules of heptadecyloxy (4-methoxyphenoxy) dimethylsilane with a molecular weight of 436.74 g/mol, a flash point of 260.5 ± 30.1 °C, and a boiling point of 507.1 ± 50.0 °C at a pressure of 760 mm-Hg. The other substances were identified and their molecular weights obtained using the GC-MS testing are shown in Figure 6.

The testing results showed that the silane on the carbon fibers were molecules containing a high carbon number and a small alkoxy group; it exhibited high hydrolysis ability despite being a dimethyl group molecule. Therefore, this study used various approaches to remove the silane from the surfaces of the carbon fibers according to the silane’s chemical and physical properties. The chemical properties of the silane were as followings: it possessed favorable hydrolysis ability and exhibited a condensation reaction approaching the point of zero charge at a pH level close to 2. Additionally, the silane showed favorable hydrolysis ability in neutral water, particularly when it was at a low concentration because OH^−^ ions contributed to its hydrolysis ability. The approach adopted based on the silane’s physical properties was to heat the silane at a temperature higher than its boiling point. Moreover, because the silane was a high-carbon-number molecule, isopropanol alcohol would not be an appropriate organic solvent for cleaning carbon fiber surfaces in the last experimental stage. Accordingly, isopropyl alcohol aqueous solution was employed as the organic solvent for cleaning the surfaces of carbon fibers, thereby removing the residual silane from the surfaces of carbon fibers.

### 4.2. SEM Analysis

#### 4.2.1. Unprocessed Carbon Fibers

**Figure 7:**
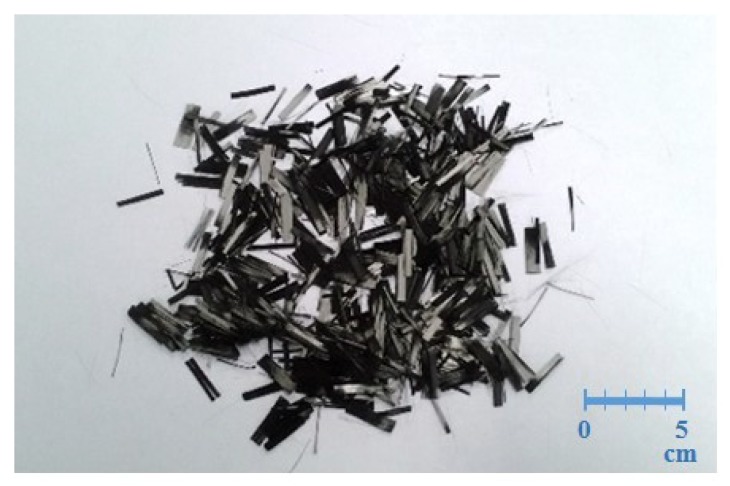
Appearance of carbon fibers.

**Figure 8:**
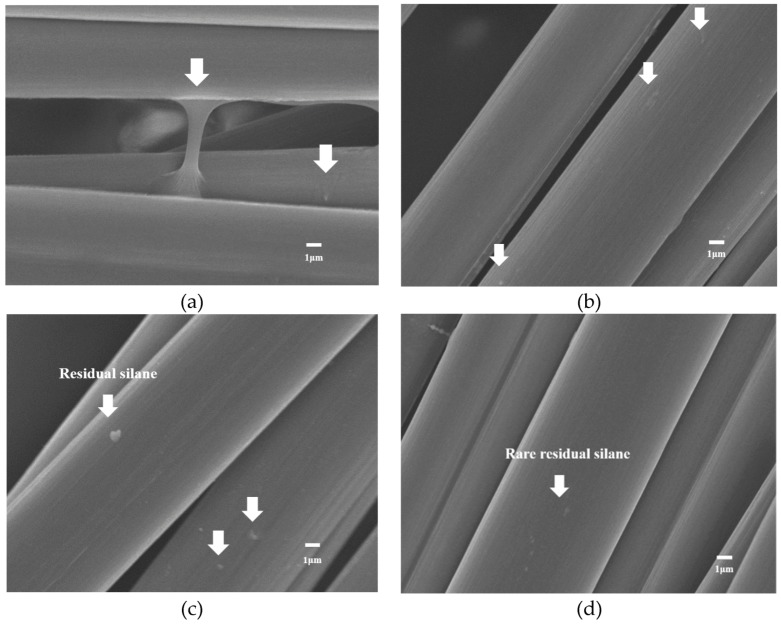

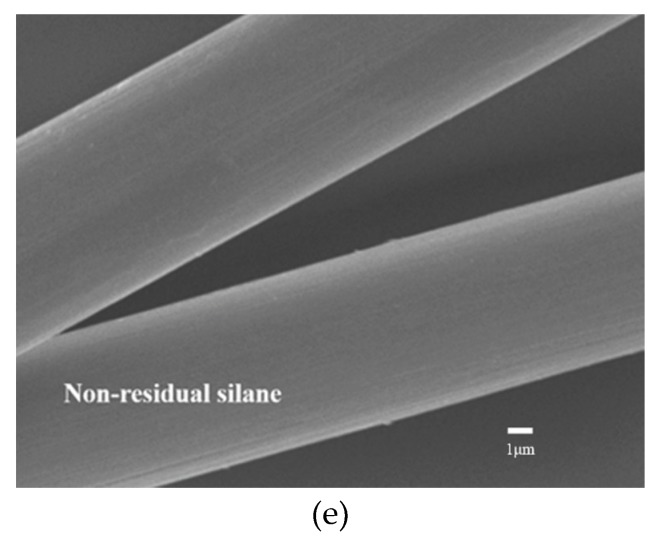
The SEM images of residual silane on short carbon fiber surface (**a**) unprocessed and using (**b**) water, (**c**) sodium hydroxide, (**d**) acetic acid, and (**e**) direct high-temperature heating.

Figure 7 shows the appearance of carbon fibers, the short carbon fibers were coupled together with the silane as the coupling agent. From the SEM analysis, there were some silane residuals for unprocessed carbon fibers shown in Figure 8a.

#### 4.2.2. Silane Removal Using a Hydrolysis Method

Based on the SEM results, silane residuals were observed on the short carbon fiber surfaces after using pure water to remove the silane. Because this method entailed reducing the concentration of silane to a low concentration, a large amount of pure water was required. Figure 8b presents SEM images of the residual silane on the short carbon fiber surfaces after pure water was used to remove the silane.

Silanes have a hydrolysis reaction in pure aqueous solution, but different types of silanes exhibit different hydrolysis abilities in water, and the hydrolysis and condensation reaction of silanes vary across temperatures. When the concentration of silane is low, the hydrolysis reaction of silane is much stronger than its condensation reaction, which is slowed under such a low-concentration condition. Both of the hydrolysis and condensation reaction of silanes in water depend on temperature. In using pure water to remove the silane from the surfaces of the carbon fibers, this study compared the reaction conditions of non-heating and heating, and the results revealed that heating to and maintaining a temperature of 100°C for 10 min produced greater removal effectiveness. Then, the short carbon fibers were removed and cleaned with an isopropyl alcohol aqueous solution before being stove-dried. Subsequently, the short carbon fibers were separated by pneumatic dispersion.

#### 4.2.3. Silane Removal Using Sodium Hydroxide

The results showed that using sodium hydroxide to remove the silane from the short carbon fiber surfaces led to silane residuals remaining on the surfaces. The purpose of using sodium hydroxide as a solvent was to increase the OH^−^ concentration in the aqueous solution and increase the degree of dissociation of water, thereby enhancing the hydrolysis reaction of the silane. Figure 8c shows SEM images of the residual silane on the short carbon fiber surfaces after removal of silane using sodium hydroxide. 

H^+^ and OH^−^ in solutions can serve as catalysts to accelerate the hydrolysis reaction of silanes [2]. To increase the concentration of OH^−^ in water, this study used sodium hydroxide to produce an aqueous solution for subsequent experiments. As described previously, silanes have a hydrolysis reaction in water; however, different types of silanes exhibit different hydrolysis abilities in water. Generally, larger alkoxy groups are associated with lower hydrolysis rates [1]. Moreover, temperature influences the hydrolysis and condensation reaction of silanes; higher temperatures enable a higher degree of dissociation of water with sodium hydroxide.

Accordingly, the present study compared with silane removal effects at various pH levels (<7, =7 and >7) environment. The testing results suggested that sodium hydroxide was more effective at removing the silane when the aqueous solution had a pH level close to 7. Therefore, we immersed the short carbon fibers in pure water with a small amount of sodium hydroxide (0.01 M) added. The greater effectiveness of silane removal was achieved when the sodium hydroxide solution was heated to 100 °C for 10–12 min. Subsequently, the short carbon fibers were removed from the solution, cleaned using an isopropyl alcohol aqueous solution, and stove-dried. After the stove-drying process, the short carbon fibers were separated using pneumatic dispersion. Overall, the residuals were still observed on the short carbon fiber surfaces after the silane removal using a sodium hydroxide solution.

#### 4.2.4. Silane Removal Using Acetic Acid

The SEM images showed that silane was barely observed on the short carbon fibers after silane removal was performed using acetic acid, indicating the high effectiveness of acetic acid (Figure 8d). Because the silane had a condensation reaction approaching the point of zero charge when at a pH of 2, indicating only a hydrolysis reaction, this study used acetic acid as a solvent. The silanes dissolve in the isopropanol alcohol and exhibit a hydrolysis reaction at a pH level between 2 and 6; moreover, the hydrolysis and condensation reactions of the silanes are related to the effect of temperatures. This study attempted to identify a test condition that would create the strongest hydrolysis reaction relative to the condensation reaction by comparing various test conditions of various acetic acid concentrations, pH levels, and temperatures. The results indicated that when acetic acid aqueous solution had a pH level of 2, silane’s condensation reaction approached the point of zero charge and exhibited only a hydrolysis reaction. Warming up the solution to and maintaining it at a temperature of 80 °C for 8–10 min led to relatively effective silane removal. Overall, only a small amount of residual silane was observed after the acetic acid aqueous solution was used to remove the silane from short carbon fiber surfaces, suggesting this to be the optimal chemical method among the three.

#### 4.2.5. Silane Removal Using High-Temperature Heating

An SEM observation suggested that nearly no residual silane remained on the short carbon fiber surfaces after heating the carbon fibers at high-temperature was used to remove the silane from the carbon fibers; Figure 8e presents the relevant SEM images. Through GC-MS testing, this study found that the silane had a flash point of approximately 260 ± 30 °C and a boiling point of 507 ± 50 °C at a pressure of 760 mm-Hg. The silanes were liquid substances, and thus this study chose to remove the observed silane from the fiber surfaces using a high-temperature furnace to burn out the silane directly. This study compared the silane-removal effectiveness in various temperature ranges, namely 500–530, 530–550, 550–570, and 570–600 °C. The results revealed that with the same heating time, residual silane was barely observed after it was heated to a temperature interval of 530–550 °C, which is higher than its boiling point, for 3 h. When the aforementioned chemical methods were compared with the physical method, which involved direct heating under high temperature, the physical method was found to be the most effective with high silane-removal effect among all the methods.

### 4.3. Pneumatic Dispersion after Silane Was Removed from Carbon Fiber Surfaces

A pneumatic disperser has a cylindrical container with multiple small vents. It can be used for a high-pressure airstream to flow out. The short carbon fibers were placed into the container, and the top of the container covered with a plastic lid owning a hole on it. The container was sealed, and one chamber has to be pumped and a high-pressure airstream go through the hole led to enormous disturbance. Hence, the carbon fibers in the container can be separated more effectively.

**Figure 9:**
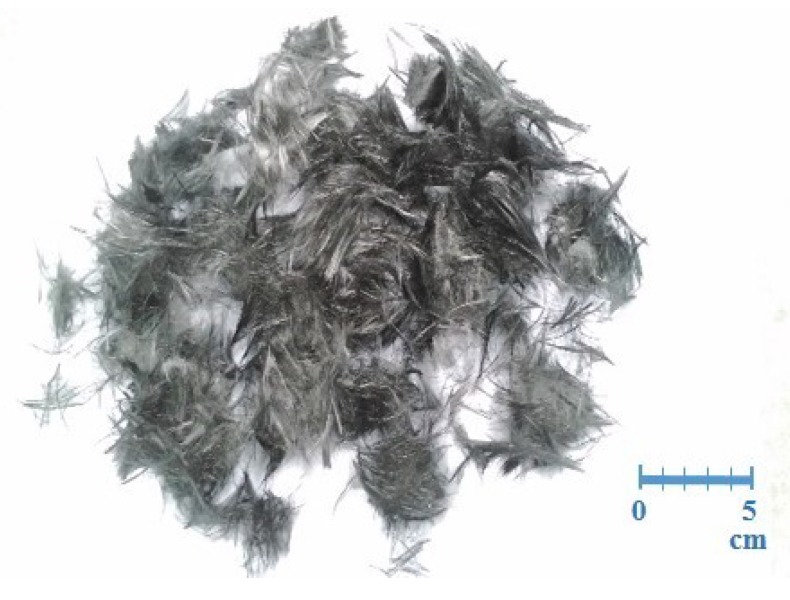
Appearance of carbon fibers after dispersion.

Before the silane was removed, the short carbon fibers adhered together through the silane as a coupling agent. Figure 9 shows the appearance of the carbon after the silane was removed and the short carbon fibers were pneumatically dispersed. Carbon fibers of 10.0 g and 24 mm were used to remove silane by the above methods; the weights of the carbon fibers after the removal of silane using different methods are shown in Table 2.

### 4.4. Compressive Testing

Mixed with an appropriate amount of short carbon fibers and water into cement, carbon-fiber-reinforced cement specimens were fabricated with compressive strengths, which were higher than the cement without the carbon fibers. The level of improvement in the overall strength of the carbon-fiber-reinforced cement varied across the different lengths of the short carbon fiber. After the silane removal and dispersion processes, the short carbon fibers formed strands, which facilitated a more uniform distribution of carbon fibers in the cement and contributed to enhancing the carbon-fiber-reinforced cement’s overall strength. 

**Figure 10:**
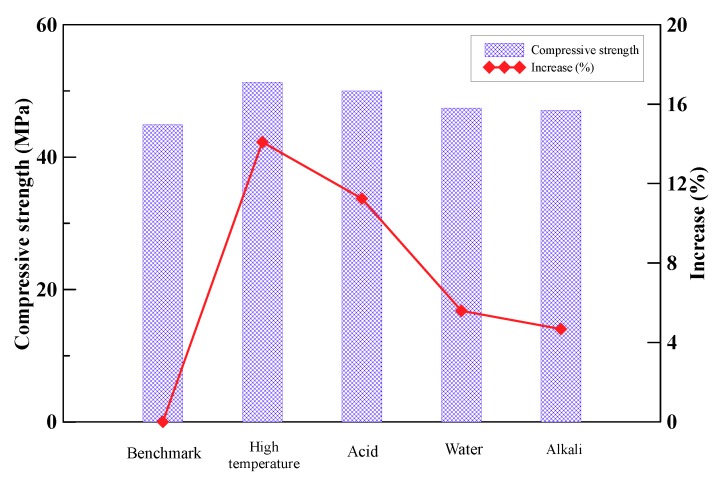
Bar chart of the compressive strength for benchmark (unprocessed), high temperature, acid solution, water, and alkali solution.

The carbon-fiber-reinforced specimens were produced after using high-temperature heating process, acid solution method, water, and alkali method to remove the silane and pneumatic dispersion to separate the short carbon fibers. The mixture proportion of the carbon-fiber-reinforced specimens was described in Section 3.3. All the carbon-fiber-reinforced specimens were put in the same condition for room temperature curing, which was between 20 °C to 25 °C. These specimens were divided into groups, each of which comprised six specimens; the compressive strengths of high-early-strength cement with unprocessed short carbon fibers added and that with dispersed short carbon fibers added and their compressive strengths are shown in Table 3. As shown in Table 4, compared with the compressive strengths of carbon-fiber-reinforced cement specimens comprising unprocessed short carbon fibers, those of carbon-fiber-reinforced cement specimens comprising short carbon fibers processed using high temperature (14.1%), acid solution (11.2%), water (5.59%), and alkali solution (4.67%) were increased. Figure 10 shows the relationships of the compressive testing for benchmark (unprocessed), high temperature, acid solution, water, and alkali solution.

### 4.5. Impact Testing

**Figure 11:**
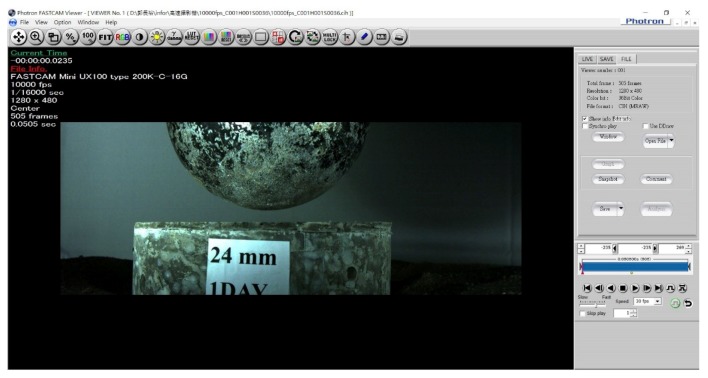
The image of benchmark specimen before the steel ball impact taken by high speed camera.

**Figure 12:**
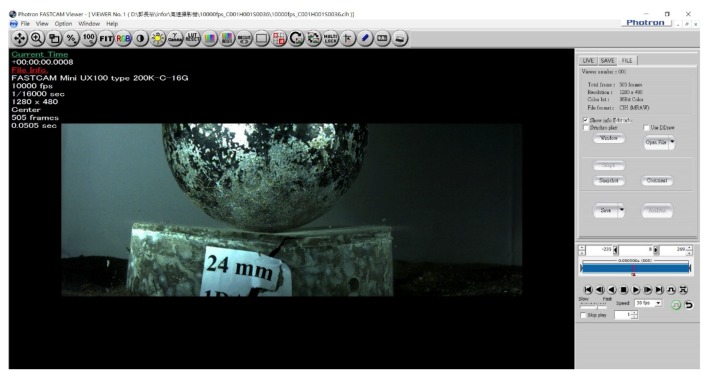
The image of benchmark specimen after the steel ball impact taken by high speed camera.

The benchmark specimen was produced by using unprocessed carbon fibers, and the compared carbon-fiber-reinforced cement specimen was produced using high-temperature heating process to remove the silane. Both specimens were using pneumatic dispersion to separate the short carbon fibers. The mixture proportion of the carbon-fiber-reinforced cement specimens was described in Section 3.3. All the carbon-fiber-reinforced specimens were put in the same condition for room temperature curing, which was between 20 °C to 25 °C. Furthermore, the high-speed camera could record evolution images of impact damage of carbon-fiber-reinforced cement specimens during impact test. Figure 11 and Figure 12 are the imaged of the benchmark specimen before and after the steel ball impact taken by high speed camera. As seen from Figure 12, the benchmark specimen was failure after the steel ball impact under the impact energy 141 J. By conducting the impact using high-speed camera, it was possible to observe and evaluate the impact resistance of carbon-fiber-reinforced cement.

For the single impact testing, using high-speed camera to obtain the impact velocity, rebound velocity and the contact duration time, then substitute the above measured data into Equation (1); the impact force of the benchmark specimen can be calculated and is shown in Table 5. 

Furthermore, the maximum impact energies of the benchmark specimen and high-temperature silane removal carbon-fiber-reinforced specimen are 141 J, and 345 J, respectively, and shown in Table 6. The impact energy of carbon-fiber-reinforced specimen with high-temperature silane removal increase 145% of the maximum impact energy compare to that of the benchmark specimen.

**Figure 13:**
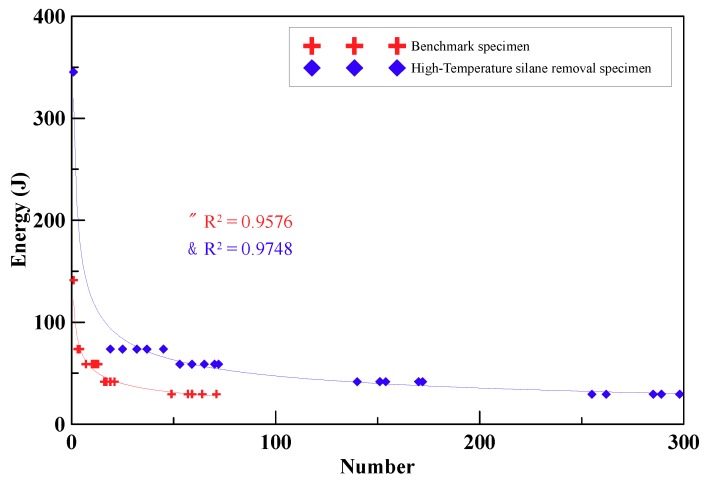
Energy–impact number curve of the benchmark and high-temperature heating processed carbon-fiber-reinforced cement specimens.

According to the repeated impact testing results, this study produced an energy–impact number curve (E–N curve) for 24-mm carbon-fiber-reinforced cement. In Figure 13, the vertical axis represents constant energies and the horizontal axis means the numbers of the impact. The specimens with 24-mm unprocessed fibers could endure 50–70 times of impacts and that with 24-mm dispersed fibers could withstand 250–290 times of impacts. As shown in Table 7, impact resistance of the specimens in the impact test added the carbon fibers after dispersion is better than those added unprocessed carbon fibers.

**Figure 14:**
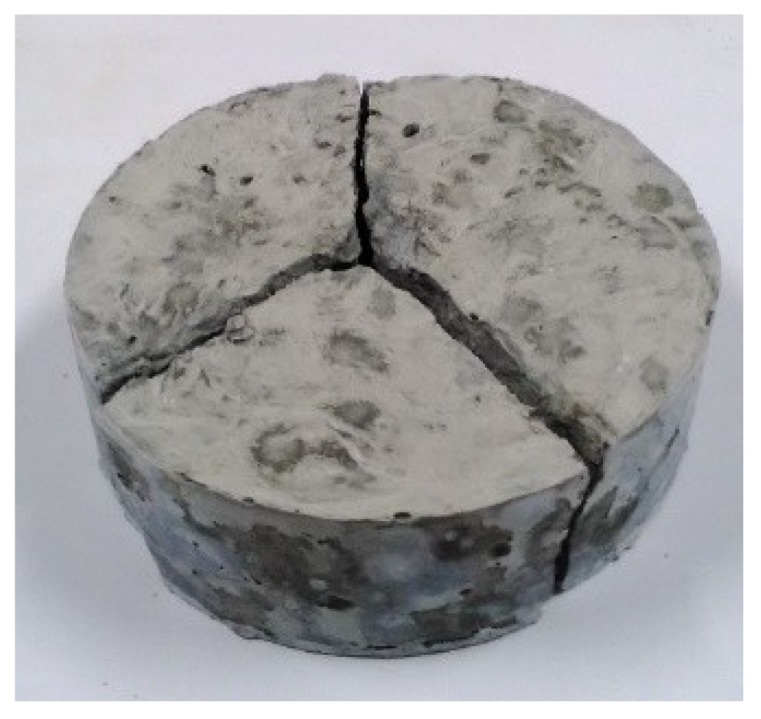
The fracture photo of the most specimen after impact test.

**Figure 15:**
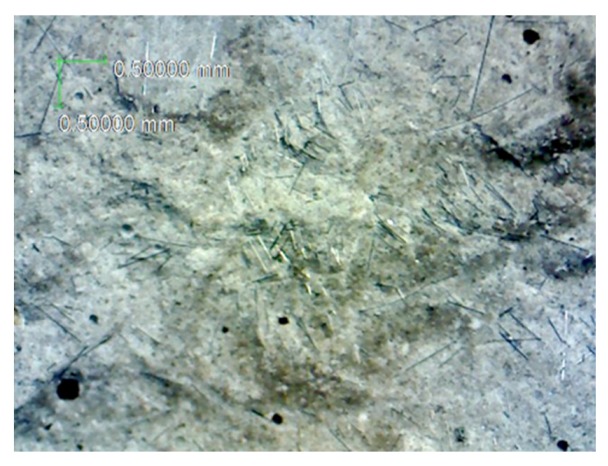
The optical microscopic photo of the specimen unprocessed carbon fibers after impact test.

**Figure 16:**
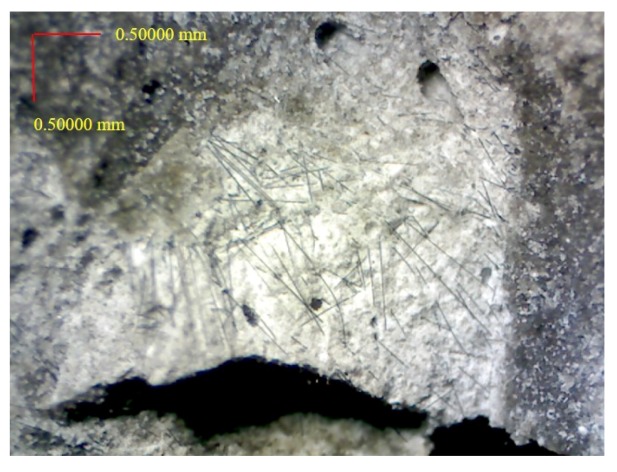
The optical microscopic photo of the specimen using high-temperature heating process after impact test.

Figure 14 is the damage photo after impact testing for most of the specimens. The specimen was equally separated into three pieces; it means that the carbon fiber filaments are uniformly distributed in the specimens. Figure 15 is the optical microscopic photo of the specimen contained unprocessed carbon fibers after impact testing. As seen from Figure 15, the carbon fiber filaments are not uniformly dispersed but some of the carbon fiber filaments are bonded together due to unprocessed carbon fibers. Figure 16 is the optical microscopic photo of the specimen with carbon fibers using high-temperature heating process after impact testing; and the carbon fiber filaments were separated and uniformly dispersed. As seen in Figure 15 and Figure 16, those carbon fiber filaments showed fracture failure; this means that the carbon fiber filaments play the role of taking tension forces under impact loading.

## 5. Conclusions

Based on the results of this study, the following conclusions can be drawn:

1. Regarding the chemical methods used for removing silane from the carbon fiber surfaces, a small amount of residual silane was observed after the silane was removed using water and a sodium hydroxide solution, whereas the residual silane was barely observed after the silane was removed using an acetic acid solution.
2. The physical method used for removing the silane from the carbon fiber surfaces involved heating the short carbon fibers to 530–550 °C, temperatures higher than the silane’s boiling point, for 3 h. Compared with the chemical methods, this direct-heating method exhibited higher effectiveness in silane removal, and residual silane was barely observed after. Based on an SEM observation, using high-temperature heating to remove the silane from the carbon fiber surfaces was the optimal method.
3. Removing the silane using high-temperature heating, acid, water, or alkali, followed by pneumatic dispersion, effectively separated the short carbon fibers. Among the four approaches, high-temperature heating demonstrated the highest effectiveness, followed by acid, water, and alkali.
4. The short carbon fibers were used after the silane removal to produce the carbon-fiber-reinforced cement specimens. These specimens of the short carbon fibers exhibited higher compressive strengths than those comprising unprocessed short carbon fibers; specifically, the improvements in compressive strengths using the various silane-removal approaches were 14.1% for high-temperature heating, 11.2% for acid, 5.59% for water, and 4.67% for alkali.
5. From the impact testing results, the highest one-time impact failure energies of the specimens with the unprocessed carbon fibers and high-temperature heating processed carbon fibers are 141.26 J and 345.45 J, respectively. In the case of the repeated impact testing, the specimen added the high-temperature heating processed carbon fibers can endure more impact arbitrarily times than that of the unprocessed carbon fibers. It means that the specimens with high-temperature heating processed carbon fibers can endure more impact energy than that of the unprocessed carbon fibers.
6. The study improved the aggregate of the short carbon fibers by a novel methodology of siliane removal accompanying with pneumatic dispersion aiming for uniformly dispersed in carbon-fiber-reinforced cement to improve its mechanical properties. Moreover, the price of the carbon fiber recently is much less than that in last two decades. Hence, this novel high-temperature heating process carbon-fiber-reinforced cement is a potential material for utilities of civil engineering infrastructure.

## Figures and Tables

**Table 1:** Composition of high-early-strength cement analyzed using X-ray fluorescence analysis.

Chemical Composition	Percentages (%)
CaO	68.4
SiO_2_	11.9
Al_2_O_3_	9.20
Fe_2_O_3_	2.90
K_2_O	1.80
TiO_2_	0.70
SO_2_	5.10
P_2_O_5_	0.50
Other	0.10

**Table 2:** The weight of carbon fibers after removal silane using different methods. (Unit: g).

Benchmark	High Temperature	Acid	Water	Alkali
10.0	9.7	9.8	9.9	9.9
	9.8	9.8	9.9	9.9
	9.8	9.8	9.9	9.9
	9.7	9.9	9.9	9.9
Average	9.75	9.83	9.90	9.90

**Table 3:** Compressive strengths of high-early-strength cement with different dispersed processes and unprocessed short carbon fibers. (Unit: MPa).

Benchmark	High Temperature	Acid	Water	Alkali
46.5	54.9	51.9	47.6	48.6
43.5	53.0	49.0	47.5	49.4
43.7	53.2	48.3	46.7	46.0
42.8	49.2	48.4	48.7	45.9
45.7	51.7	50.6	47.1	45.7
45.4	45.6	51.8	47.0	46.7

**Table 4:** The comparisons of the compressive strength for benchmark and different processes carbon-fiber-reinforced cement specimens.

Process Method	Benchmark	High Temperature	Acid	Water	Alkali
Compressive strength (MPa)	44.9	51.3	50.0	47.4	47.0
Increase (%)	-	14.1	11.2	5.59	4.67

**Table 5:** The impact force of the benchmark fiber-reinforced cement specimen.

Specimen	Numbers	Status	Mass (kg)	V_1_ (m/s)	V_2_ (m/s)	Contact Time Duration (s)	Force (kN)
24 mm	1	Failure	7.2	5.94	0.5	0.0179	11.23

**Table 6:** The comparisons of the maximum impact energy for benchmark and high-temperature silane removal carbon-fiber-reinforced cement specimens.

Process Method	Benchmark	High-Temperature Silane Removal
Impact energy (J)	141	345
Increase (%)	-	145

**Table 7:** The comparisons of the impact number for high-early-strength cement between high-temperature dispersed and unprocessed carbon fibers (24 mm).

Carbon Fiber	Impact Energy (J)		73.6	58.9	41.7	29.4
Unprocessed(Benchmark)	Impact number		4	13	21	71
			4	12	21	64
			4	11	19	59
			3	10	17	57
			3	7	16	49
		**Average**	3.8	10.6	18.8	60
High- temperature heating (Remove Silane)	Impact number		32	70	154	255
			19	53	170	285
			37	72	140	298
			25	59	151	262
			45	65	172	289
		**Average**	31.6	63.8	157	278
**Impact number increase (%)**			732	502	735	363

## References

[1] Matinlinna J.P., Lung C.Y.K., Tsoi J.K.H. (2018). Silane adhesion mechanism in dental applications and surface treatments: A review. Dent. Mater..

[2] Brinker C. (1988). Hydrolysis and condensation of silicates: Effects on structure. J. Non-Crystalline Solids.

[3] Han B., Zhang L., Zhang C., Wang Y., Yu X., Ou J. (2016). Reinforcement effect and mechanism of carbon fibers to mechanical and electrically conductive properties of cement-based materials. Constr. Build. Mater..

[4] Chen M., Gao P., Geng F., Zhang L., Liu H. (2017). Mechanical and smart properties of carbon fiber and graphite conductive concrete for internal damage monitoring of structure. Constr. Build. Mater..

[5] Feng H., Le H.T.N., Wang S., Zhang M.-H. (2016). Effects of silanes and silane derivatives on cement hydration and mechanical properties of mortars. Constr. Build. Mater..

[6] Sassani A., Ceylan H., Kim S., Gopalakrishnan K., Arabzadeh A., Taylor P.C. (2017). Influence of mix design variables on engineering properties of carbon fiber-modified electrically conductive concrete. Constr. Build. Mater..

[7] Lu M., Xiao H., Liu M., Li X., Li H., Sun L. (2018). Improved interfacial strength of SiO 2 coated carbon fiber in cement matrix. Cem. Concr. Compos..

[8] Chung D. (2000). Cement reinforced with short carbon fibers: a multifunctional material. Compos. Part B: Eng..

[9] Yang Y. (2001). Methods Study on Dispersion of Fibers in CFRC. Cement Concrete Res..

[10] Wang Z., Gao J., Ai T., Jiang W., Zhao P. (2014). Quantitative evaluation of carbon fiber dispersion in cement based composites. Constr. Build. Mater..

[11] Larson B., Drzal L., Sorousian P. (1990). Carbon fibre-cement adhesion in carbon fibre reinforced cement composites. Compos..

[12] Xu Y., Chung D. (1999). Carbon fiber reinforced cement improved by using silane-treated carbon fibers. Cem. Concr. Res..

[13] Lavagna L., Musso S., Ferro G., Pavese M. (2018). Cement-based composites containing functionalized carbon fibers. Cem. Concr. Compos..

[14] Cui H., Jin Z., Zheng D., Tang W., Li Y., Yun Y., Lo T.Y., Xing F. (2018). Effect of carbon fibers grafted with carbon nanotubes on mechanical properties of cement-based composites. Constr. Build. Mater..

[15] Capela C., Oliveira S., Pestana J., Ferreira J. (2017). Effect of fiber length on the mechanical properties of high dosage carbon reinforced. Procedia Struct. Integr..

[16] Li V.C., Obla K.H. (1994). Effect of fiber length variation on tensile properties of carbon-fiber cement composites. Compos. Eng..

[17] Gao J., Sha A., Wang Z., Hu L., Yun D., Liu Z., Huang Y. (2018). Characterization of carbon fiber distribution in cement-based composites by Computed Tomography. Constr. Build. Mater..

[18] Wang C., Jiao G.-S., Li B.-L., Peng L., Feng Y., Gao N., Li K.-Z. (2017). Dispersion of Carbon Fibers and Conductivity of Carbon Fiber-Reinforced Cement-Based Composites. Ceram Int..

[19] Zhou J., Qian S., Ye G., Çopuroğlu O., Van Breugel K., Li V.C. (2012). Improved fiber distribution and mechanical properties of engineered cementitious composites by adjusting the mixing sequence. Cem. Concr. Compos..

[20] Gao J., Wang Z., Zhang T., Zhou L. (2017). Dispersion of carbon fibers in cement-based composites with different mixing methods. Constr. Build. Mater..

[21] Yang Y., Deng Y. (2018). Mechanical properties of hybrid short fibers reinforced oil well cement by polyester fiber and calcium carbonate whisker. Constr. Build. Mater..

[22] Wang C., Li K.-Z., Li H.-J., Jiao G.-S., Lu J., Hou D.-S. (2008). Effect of carbon fiber dispersion on the mechanical properties of carbon fiber-reinforced cement-based composites. Mater. Sci. Eng. A.

[23] Tabatabaei Z.S., Volz J.S., Keener D.I., Gliha B.P. (2014). Comparative impact behavior of four long carbon fiber reinforced concretes. Mater. Des..

[24] Ulas M.A., Alyamaç K.E., Ulucan Z.C. (2018). Development of nomogram for the practical mix design of steel fiber reinforced concrete. Constr. Build. Mater..

[25] Safiuddin M., Yakhlaf M., Soudki K. (2018). Key mechanical properties and microstructure of carbon fibre reinforced self-consolidating concrete. Constr. Build. Mater..

[26] Newcomb B.A. (2016). Processing, structure, and properties of carbon fibers. Compos. Part A Appl. Sci. Manuf..

[27] ASTM C109/C109M-02 (2016). Test Method for Compressive Strength of Hydraulic Cement Mortars (Using 2-in. or [50-mm] Cube Specimens).

[28] ASTM D 5628 (2018). Standard Test Method for Impact Resistance of Flat, Rigid Plastic Specimens by Means of a Falling Dart (Tup or Falling Mass).

